# The influence of provider payment mechanisms on TB service provider behavior in Indonesia: insights from National Health Insurance data and provider perspectives

**DOI:** 10.3389/fpubh.2025.1396596

**Published:** 2025-07-09

**Authors:** Meghan O'Connell, Firdaus Hafidz, Sarah Saragih, Cheryl Cashin, Aditia Nugroho, Laurel Hatt, Yuli Farianti, Ackhmad Afflazier, Imran Pambudi

**Affiliations:** ^1^Results for Development, Washington, DC, United States; ^2^Department of Health Policy and Management, Faculty of Medicine, Public Health, and Nursing, Universitas Gadjah Mada, Yogyakarta, Indonesia; ^3^Results for Development, Jakarta, Indonesia; ^4^Ministry of Health, Jakarta, Indonesia

**Keywords:** National Health Insurance, provider payment, referral, tuberculosis, Indonesia

## Abstract

**Background:**

The impact of provider payment mechanisms under Indonesia’s National Health Insurance (NHI) scheme on healthcare providers’ behavior–particularly in tuberculosis (TB) service delivery– remains underexplored. This study examines the consequences of provider payment incentives on TB service provider behavior.

**Methods:**

A mixed-methods study was conducted using quantitative analysis of NHI claims data from 2015 to 2016 and qualitative data from focus group discussions with healthcare providers—22 primary care facilities and 14 hospitals across five provinces-. Quantitative analysis examined TB service utilization patterns, assessed referral appropriateness based on case complexity, and claim of TB services. Qualitative data were thematically analyzed to explore factors influencing provider decision-making in the context of payment mechanisms and service delivery under the NHI scheme.

**Results:**

Findings indicate that primary care facilities refer a high proportion of TB cases to secondary-level care, even for uncomplicated cases (81% of 782 visits). Secondary care recorded significantly more TB visits than primary care (5,249 vs. 1,094 visits), resulting in an estimated USD 14.1 million in potentially avoidable costs for the NHI program. If these cases had been managed at the primary level, potential cost savings could have been substantial. Qualitative analysis revealed that provider referral decisions were influenced by capitation-based payment structures, limited diagnostic tools, absence of dedicated TB rooms, lack of provider capacity, patient preferences, financial incentives favoring more profitable diseases, and providers’ social ties. The high rate of up-referrals may negatively impact service quality and TB treatment outcomes.

**Conclusion:**

Current provider payment mechanisms under NHI contribute to inefficiencies in TB service delivery by incentivizing unnecessary referrals to secondary care. Optimizing payment methods and strengthening implementation by addressing weak provider capacity at the primary care level could enhance incentives for primary-level management of TB cases, improving cost-effectiveness and service quality.

## 1 Introduction

Tuberculosis (TB) remains a major public health challenge in low- and middle-income countries (LMICs), where financial and structural barriers hinder access to timely diagnosis and treatment (1). Integrating TB services into National Health Insurance (NHI) schemes has been recognized as a strategy for ensuring sustainable financing and improving access to TB care. The Indonesian NHI Scheme, *Jaminan Kesehatan Nasional* (JKN) was launched in 2014. The scheme together with the National TB program by the Ministry of Health includes TB diagnosis and treatment services as part of its benefits package. JKN-covered TB patients are served across both public and private providers at primary and secondary care levels. The program aims to reduce financial barriers to care and improve access to TB services across public and private healthcare providers. Under JKN, purchasing arrangements are designed to provide coverage to the entire population, with enrollees grouped into two categories: premium assistance beneficiaries—funded by the national or local governments and targeting the poor—and non-premium assistance beneficiaries, which include salaried workers, informal workers, and non-employed individuals (2, 3).

Several countries have implemented NHI-funded TB services with varying degrees of success (4). The effectiveness of TB service delivery under the NHI framework is influenced by provider payment mechanisms, which shape provider behavior and treatment pathways (5). Within Indonesian NHI scheme primary-care facilities receive a fixed monthly capitation, while hospitals are reimbursed via bundled Indonesia Case-Based Groups (INA-CBGs); capitation offers no additional reward for active case finding (6), whereas INA-CBGs may encourage hospitals to retain uncomplicated cases (5, 7, 8).

NHI-funded TB services affect access, cost, and care-seeking behavior, with findings from Taiwan, India, China and Vietnam showing disparities in patient pathways, reimbursement rates, and financial protection (9–13). While these studies inform the financial impact of NHI on TB patients, fewer have investigated how provider payment mechanisms shape provider behavior. This is particularly relevant in Indonesia, where the JKN uses capitation at the primary level and case-based payments in hospitals, potentially influencing referral patterns and case retention (5–7). This study addresses a critical evidence gap by exploring the effects of JKN’s payment mechanisms on TB service delivery.

Despite the increasing focus on strategic purchasing in LMICs, limited research has examined how payment mechanisms impact TB service delivery within Indonesia’s NHI framework. Understanding these dynamics is critical for optimizing financing strategies and strengthening primary care engagement in TB management. This study examines the consequences of provider payment incentives on TB service delivery under Indonesia’s NHI, focusing on referral dynamics between primary health care (PHCs) and hospitals. Specifically, we investigate: (i) how TB diagnosis, referral, and treatment patterns are shaped by payment mechanisms using national NHI claims data; and (ii) how provider decisions regarding referral and treatment are influenced by payment incentives through qualitative analysis. The findings will provide insights into how strategic purchasing can be optimized to encourage efficient TB service delivery and strengthen primary care engagement in TB management.

## 2 Materials and methods

### 2.1 Study setting

Indonesia has one of the highest TB burdens globally, ranking second after India (1). TB remains the leading cause of death from communicable diseases in Indonesia, with an estimated 824,000 incident cases and 93,000 TB-related deaths in 2019 (14). Although TB mortality declined by 19% between 2007 and 2017, the disease remains a significant public health challenge (15). Beyond its health burden, TB is also financially catastrophic for many patients due to treatment-related expenses and loss of income (16).

The key challenge in TB control is the high number of missing cases—cases that are either undiagnosed or diagnosed but not reported. An inventory study published in the 2018 WHO Global TB Report found that 18% of TB cases in Indonesia were truly missing (not detected or reported), while 29% were diagnosed but not reported (17). Under-detection and under-reporting are particularly concerning in private and secondary care settings, where TB notification rates remain low (18).

### 2.2 Study design

This study employed a sequential mixed-methods approach, integrating quantitative and qualitative analyses. The quantitative phase was conducted first, followed by the qualitative phase, which provided deeper insights into the patterns observed in the quantitative data.

### 2.3 Study population

The study population for quantitative analysis consisted of patients covered under JKN scheme who were diagnosed with TB in 2015–2016. Covering services delivered in PHC facilities and hospitals. For the qualitative analysis, we sought the perspective of frontline and managerial stakeholders in both public and private sectors: primary care, and hospital involved in TB service delivery. The study received ethical approval from the Universitas Gadjah Mada Research Ethics Committees (KE/FK/0934/EC/2019).

### 2.4 Sampling method

We utilized Indonesia’s National Health Insurance (NHI) Agency, *Badan Penyelenggara Jaminan Sosial Kesehatan* (BPJS-K) sample dataset −1% representative sample of 2015–2016 NHI claims for the quantitative analysis. The dataset includes capitation-based claims, non-capitation fee-for-service claims, and hospital claims under bundled, case-based payments (INA-CBG claims). For the qualitative analysis, we employed purposive maximum-variation sampling. Five high-burden provinces—West Java, Banten, East Java, South Sulawesi, and North Sumatera— were selected based on the following criteria: the presence of GeneXpert for TB rapid molecular diagnostic, and the involvement of private providers in TB program services. Within those provinces we chose nine urban and rural districts drawn from the Ministry of Health’s priority list and accessibility.

### 2.5 Data collection

**Figure 1:**
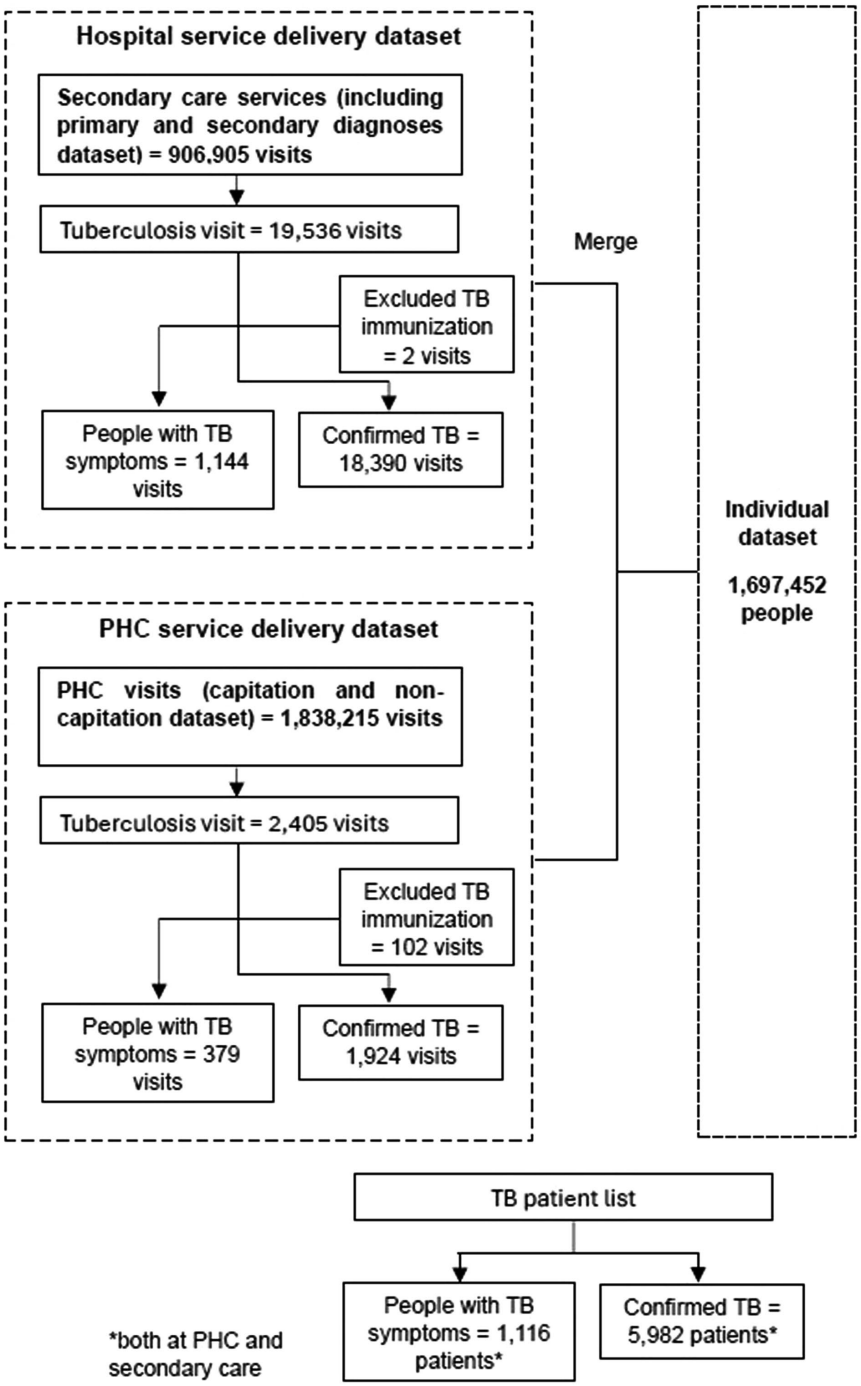
Process to identify TB patient list.

We obtained a nationally representative 1% sample of 2015–2016 JKN claims released by BPJS-K in February 2019 (BPJS Kesehatan, 2019). The dataset included three components: (1) an individual-level dataset to explore patient characteristics, i.e., demography and insurance segment; (2) a primary care service delivery dataset to explore characteristics of TB services at the primary care level; (3) a secondary care service delivery dataset to explore TB services in hospitals. We identified TB cases by searching using keyword “tuberculosis” in ICD-X description of diagnosis, and removing Z23.2 (TB immunization). For the final data set, we added the secondary care dataset to the primary care dataset and merged them with the individual dataset to identify patients with TB diagnoses. Subsequently, we collapsed by patient ID to obtain a list of TB patients who utilized services (Figure 1). A detailed list of variables utilized in the analysis can be found in Supplementary material 1.

To explore provider behavior and financing in greater depth, we held Focus Group Discussions (FGDs) between August and September 2019. The FGDs as qualitative data collection was completed on behalf Indonesia Ministry of Health and considered assessment at a national level. The Ministry of Health (MoH) led each session, while our research team facilitated the conversation and took analytic field-notes. All FGDs were recorded, a notetaker was assigned for each session, and each FGD was transcribed. Systematic coding was carried out in Excel to identify and organize themes and characteristics that emerged from the data. Participants were grouped by facility type to encourage focused discussion and peer validation of experiences. Focus group discussions were carried out at 40 health facilities, including 22 primary care facilities (12 public health clinics, eight private clinics, two private general practitioners), 14 hospitals (seven public hospitals, seven private hospitals), two laboratories (one public and one private lab), and two private pharmacies. We include private pharmacies and laboratories because of their important role in TB service delivery, especially testing and treatment in the private sector (19). In total, FGDs were attended by 141 participants. Data triangulation was conducted comparing information between private primary care facilities and public primary care facilities, as well as between primary care and secondary care facilities. In addition, triangulation of sources was done through data provided by health facilities.

### 2.6 Data analysis

#### 2.6.1 Quantitative analysis

Descriptive analysis using counts and percentages was conducted to examine the characteristics of TB patients and service use across primary and secondary care. We performed cross-tabulations to explore three key relationships: (1) the distribution of presumptive and confirmed TB patients and visits by level of care; (2) referral patterns from primary care, including referral status and destination (public vs. private hospitals); and (3) from the hospital perspective, the source of referrals and the complication status of referred cases. Each analysis was stratified by the type of primary-care provider.

First, to distinguish between people with TB symptoms and confirmed TB patients, we used the ICD-10 code of Z03.0 to identify cases of presumptive TB as indicated in MoH regulation no. 76 (2016) on Indonesian Case Based Groups Guidelines for National Health Insurance implementation (20). Second, healthcare providers were classified into primary care and secondary care providers. A variable was also created to identify complicated and uncomplicated TB in primary and secondary care facilities to study whether the referral was conducted following the regulation which stipulates that primary care is responsible for treating TB without complication (21). The variable to indicate complicated or uncomplicated TB was generated by extracting the ICD-10 codes for primary and secondary diagnoses of TB cases. The categorization of cases was done by a clinician SME that was part of the research team, using the general practitioner’s competency standard guidelines on TB diagnoses treated in primary care compared to those that should be referred for secondary care (21). This classification was generated by extracting the ICD-10 codes for primary and secondary diagnoses of TB cases.

The unit of measurement for referral analysis was the number of visits rather than the number of patients, to better capture the service volume associated with TB care across different provider levels. The STROBE checklist was utilized (see Supplementary material 3).

#### 2.6.2 Qualitative analysis

To ensure rigor and transparency, we adhered to the Consolidated Criteria for Reporting Qualitative Research (COREQ) in designing, conducting, and reporting (Supplementary material 2). We employed thematic analysis for qualitative data analysis, using a deductive coding approach based on pre-existing themes derived from the literature and our research questions. Themes covered included TB services, including referral behavior, referral patterns, referral decision-making, incentives or reasons for referring and retaining patients, and consequences of referral, including the shifting of patients and costs. A semi-structured guide with open-ended prompts encouraged detailed, context-rich responses within each theme.

The unit of analysis was the health facility, representing type of facility (e.g., primary care, hospital administrators), and ownership (e.g., public, private). To ensure the robustness of our findings, we used triangulation, comparing qualitative insights with quantitative data sourced from BPJS-K sample data. Any disagreements during the coding process were resolved through team discussions, where we worked together to reach a consensus, guided by the research objectives to ensure consistency in our analysis.

## 3 Results

### 3.1 Basic characteristics of TB patients

A merge dataset of 5,982 TB patients receiving care at hospitals and primary care facilities revealed that 58% were male and the majority were of productive age (15–64 years). Notably, 34% of the patients were enrolled under the employee scheme, while 50% were registered at public PHCs, indicating the central role of public PHCs in TB service delivery. As shown in Table 1, the majority was in employee scheme categories (34%). In addition, 50 % of the TB patients were registered at a public PHC.

**Table 1:** The characteristics of TB cases (number of TB patients) receiving care at health facilities (unweighted).

Description	Freq.	(%)
Total	5,982	(100)
Sex		
Male	3,450	(58)
Age		
Up to 5 years	526	(9)
6 to 16	597	(10)
17 to 65	3,940	(66)
Over 65 years	919	(15)
Segment		
Premium assistance beneficiaries	1,784	(29)
Informal	1,781	(30)
Employee	2,007	(34)
Non-employee (employer, pension)	410	(7)
Health facility registered		
Public PHC	2,973	(50)
Private clinics	1,727	(29)
Private GP	1,282	(21)

### 3.2 Utilization of presumptive and clinical TB services: higher visits in secondary care

The number of patients and visits for TB services were much higher in secondary care compared to primary care for both people with TB symptoms and confirmed TB cases. Among all visits for TB services, claims data analysis showed that there were 2,303 (10.55%) visits in primary care facilities, including for people with TB symptoms and confirmed TB. This is compared to 19,534 visits (89.45%) in secondary care facilities for people with TB symptoms and confirmed TB (see Table 2). The number of people with TB symptoms and confirmed TB patients (as opposed to visits) in primary care was also less than in secondary care.

**Table 2:** Overall people with TB symptoms and confirmed TB utilization by location (unweighted).

Health facilities	Number of people with TB symptoms	Number of visits by people with TB symptoms	Patients with confirmed TB	Number of visits by people with confirmed TB
Primary care	280	379	1,094	1,924
Secondary care	848	1,144	5,249	18,390

TB patients had more visits in secondary care compared to primary care during treatment. The number of visits at secondary care facilities for patients with confirmed TB and people with TB symptoms were 3.5 and 1.3. At the primary care level, the average number of visits was 1.8 and 1.4 for people with confirmed TB cases and symptoms, respectively. The result shows that secondary care has higher utilization for TB services compared to primary care in Indonesia.

### 3.3 Referral behavior in the TB program

Primary care providers tend to refer patients to secondary care for TB diagnosis and treatment. One-third of all primary-level visits for people with TB symptoms were referred to secondary care facilities for diagnosis; 62% (78 of 125) of these referrals were made by private clinics and general practitioners (see Table 3).

**Table 3:** Referral status of people with TB symptoms at primary care facilities (unweighted, *N* = 375, 4 missing).

Referral status	Public PHC		Private PHC		Total^*^	
	(*n* = 230)		(*n* = 145)		(*n* = 375)	
	*n* (% of column)		*n* (% of column)		*n* (% of column)	
Not referred	183	(80)	67	(46)	250	(67)
Referred	47	(20)	78	(54)	125	(33)
To Public Hospital	39	(83)	33	(42)	72	(58)
To Private Hospital	8	(17)	45	(58)	53	(42)

^*^Data indicates # of PHC visits of people with TB symptoms.

Private clinics play a pivotal role in vertical referral by primary care providers. Almost half of the confirmed TB cases were referred by primary care providers to secondary care (42%) and most of them were identified as uncomplicated (637 out of 782 visits). This was primarily driven by private clinics, which referred to 58% of their confirmed TB cases. Public PHC referred closer to 1 out of every 4 (27%) of their confirmed TB patients to secondary care for treatment (see Table 4).

**Table 4:** Referral and complication status of confirmed TB in primary care (unweighted, *N* = 1,872, 52 missing).

Referral and complication status	Public PHC		Private PHC		Total^*^	
	*n* (% of column)		*n* (% of column)		*n* (% of column)	
Not referred	726	(73)	364	(42)	1,090	(58)
Uncomplicated	630	(87)	312	(86)	942	(86)
Complicated	96	(13)	52	(14)	148	(14)
Referred for treatment	273	(27)	509	(58)	782	(42)
Uncomplicated	233	(85)	404	(79)	637	(81)
Complicated	40	(15)	105	(21)	145	(19)

^*^Data indicates # of PHC visits of confirmed TB patients.

Only a few of the private clinics interviewed offered TB diagnosis or treatment services. Insights from the qualitative interviews and incentive mapping analysis infer that the overall trend of referring TB patients to secondary care was at least in part due to the absence of diagnostic testing tools in primary care. One private clinic said that they were hindered from providing TB services and cited the absence of a separated TB room and a lack of capacity, which prompted the clinic to stop TB service provision to avoid the risk of exposure and infection for staff and other clinic patients. The exemplar quote below reflects this sentiment, which was a theme among the private clinics interviewed.

“*We do not provide TB care, first because we do not have sufficient facilities, and we have no capacity since we do not get any training. Before, we had a TB service, but we do not have a separate room for TB, so if we mixed them with other patients, it might infect other patients. So, we decided to discontinue the service*.” (Private clinic 2)

When compared to public primary care facilities, private facilities tended to refer presumptive TB cases to secondary care facilities more frequently for diagnosis. Public primary care is also equipped with sufficient diagnostic tools and information systems for specimen transportation for TB laboratory examinations (SITRUST) to send the sputum samples for GeneXpert testing. This theme is exemplified in the following interview excerpt:

“*According to the contract, we should refer to Public PHC, but it is also left to the patient [to decide]. Sometimes, the patient does not have any patience; they want to be referred for chest radiographs directly. So, we referred them to the hospital, but afterward they continued treatment at the hospital. It’s around 30% of total patients referred to Public PHC, the remaining are sent to hospital*.” (Private Clinic 2)

Several private facilities demonstrated that the referral location was decided following the patient’s preference despite MoH regulations which indicate that uncomplicated pulmonary TB should be managed in primary care. Providers indicated that the ease of referral and the patient’s perception that a hospital can be a one-stop service, contributed to a high rate of referrals to secondary care.

“*Before implementation of Time Age Complication Comorbidity (TACC, a set of criteria used for evaluating patients with TB and determining whether referral is necessary, which is required for Primary Health Facilities (FKTP) that cooperate with the Social Security Agency for Health), we referred to hospitals more. Hospitals have sufficient lab facilities, so sputum, thorax rontgen, HIV test, and glucose test can be done in one day*.” (Private Clinic 4)

Paying for TB services as part of the capitation payment might also discourage private sector providers from providing TB services or create confusion about the roles and responsibilities of public vs. private sector providers vis-a-vi TB diagnosis and treatment. For example, one independent practitioner chose to focus on treating non-communicable diseases, which are a more “profitable disease,” and preferred to allot TB services to public primary care facilities.

“*Oh, treatment should be conducted in Public PHC, because I cannot reach out to the patient. Besides, I want to focus on treating non-communicable disease patients”* (Private Clinic 2)

Many private primary care providers indicated that, if stronger financial incentives existed, they would be indeed willing and able to increase TB service provision. Such weak incentives are apparent when private facilities indicate that the low capitation amount does not encourage case finding, case notification, or retention of resource-intensive TB patients at the primary care level for diagnosis or treatment. A practitioner explained the existing weak incentives further:

“*We cannot do screening and monitoring [for] our patients*” (Private Clinic 1)

Social ties play a significant role in referrals made by public PHC. Results showed that providers that did refer patients to the public PHC tended to have informal relationships with staff which contributed to easier coordination through informal social and cultural practices (i.e., WhatsApp) of communication as quoted in the following:

“*We do not report the case [to the national TB reporting system]. If we refer to hospital, we contact the doctor, just to inform [them] that our patient is referred to that hospital*” (Private Clinic 2)

### 3.4 Consequences of provider referral behavior

The high rate of referrals and treatment of uncomplicated TB in secondary care led to much higher expenses for the NHI Program than if those cases were treated in primary care. Meaning the bulk of the excess costs were driven by TB cases that were within the clinical purview of primary care practitioners. A similar trend was also found in the secondary care dataset for outpatient hospital utilization (see Table 5). Of confirmed TB cases referred to secondary care facilities, the number of uncomplicated TB visits was almost double that of complicated TB. The most significant proportion of referred uncomplicated TB cases was found in public hospitals (56%), followed by private hospitals (42%) and private specialist clinics (2%). Outpatient care for uncomplicated TB in secondary care cost the National Health Insurance Program an estimated 188 billion rupiahs (14.1 million USD) [average exchange rate in 2016:1 USD equal to 13,307 IDR (22)].

**Table 5:** Referral and complication status of TB confirmed cases (unweighted, *N* = 18,362, 28 missing).

Treatment site/referred and complication status	Private specialist clinic		Public Hospital		Private Hospital		Total	
	*n* (% of row)		*n* (% of row)		*n* (% of row)		*n* (% of row)	
Self-referral	1	(0)	225	(73)	81	(26)	307	(100)
Uncomplicated	-	(0)	41	(73)	15	(27)	56	(100)
Complicated	1	(0)	184	(73)	66	(26)	251	(100)
Referred from other	339	(2)	10,723	(59)	6,993	(39)	18,055	(100)
Uncomplicated	273	(2)	6,457	(56)	4,883	(42)	11,613	(100)
Complicated	66	(1)	4,266	(66)	2,110	(33)	6,442	(100)

The shifting of service to primary care will be needed, as secondary care lacks capacity and willingness to conduct outreach and monitoring of the patient as observed during the interview.

“*We can only wait until the loss-to-follow-up patient come” – no home visit* (Private hospital 1)

“*It is not efficient if we have to do a home visit, better to have a partnership with Civil Society Organization (CSO that play an important role in supporting the implementation of the program and ensuring continuation of TB treatment) for [conducting] the outreach activity*” (Private hospital 5)

## 4 Discussion

This study highlights key challenges in TB service delivery under Indonesia’s National Health Insurance (NHI) scheme. A high number of TB patient visits to hospitals indicated a shift from PHCs to secondary care. Several factors contributed to this pattern, including the absence of diagnostic testing tools, lack of dedicated TB rooms, limited provider capacity, patient preferences, provider incentives for more profitable diseases, low capitation payments at PHCs, and social ties between providers. Raising the effective reimbursement rate for uncomplicated TB in rural China has been linked to better affordability and higher treatment uptake, underscoring the value of aligning payment with first-line care (23). This referral behavior has led to increased hospital utilization and higher care costs, raising concerns about system efficiency. These findings underscore the urgency of reforming provider payment mechanisms and strengthening PHC capacity to ensure more effective and sustainable TB care delivery.

The NHI data underscored those individuals presenting symptoms of TB received less than two visits, encompassing both primary and secondary care settings. Moreover, TB patients attending outpatient appointments, regardless of care level, experienced fewer than 4 encounters throughout their treatment course. Notably, the frequency of visits prior to TB confirmation was lower than what was reported in prior research, with an average of 2.56 pre-diagnostic visits (24) and a median of 6 pre-diagnostic visits (25). However, when considering the WHO guidelines for TB treatment, which recommends a treatment duration of six to 8 months (26), it becomes evident that four visits are insufficient for adequate care of TB patients.

Our findings show that referrals for presumptive and uncomplicated TB still flow mainly to hospitals, indicating that the horizontal-referral policy is not working as intended. The MoH Clinical Practice Guidelines for Physicians in PHC, state that uncomplicated pulmonary TB should be managed at the primary care level (21). Facilities that do not have adequate capacity are expected to refer patients horizontally to the better equipped public PHC -not to hospitals-. By September 2022 the government had deployed 1,812 GeneXpert machines to 1,683 facilities −925 of them public PHC-covering 500 (97%) of districts (27, 28). Persisting hospital referral therefore points to a mix of limited capacity and misaligned payment incentives in primary care, especially in the private sector.

The private sector is pivotal at the start of the TB pathway. In Vietnam, half of the patients initially sought care from private health-care providers, including private physicians and pharmacies (29). Similarly, in Indonesia, a substantial percentage initially sought care at private clinics, particularly those residing in rural districts and with lower educational levels (24). This fact is described by previous studies, that only a few TB patients encountered adequate diagnostic capacity in the private primary facilities (19, 25, 30). Our findings support several earlier studies, identifying that the referral behavioral trend to secondary care is due to lack of capacity (31), patient preferences, and long waiting time (32). In addition, our study also confirmed existing studies finding that a lack of financial incentives (i.e., the capitation payment) could motivate primary care facilities to excessively up-refer the patient (5, 18, 25, 33–35).

Strengthening primary-care capacity and realigning incentives is therefore crucial. Practical options include: (i) through imposing mandatory notification (36), (ii) by reinforcing public-private mix networks, which showed positive outcomes in Pakistan, India, and Philippines (37–39), (iii) providing Directly Observed Treatment Short-Course (DOTs) training to private primary providers (40), (iv) ensuring the supply readiness of the private sector by giving more subsidies to the private sector in relation to TB diagnosis, (v) giving additional incentives by changing payment mechanisms (5, 18). Implemented together, these measures could reduce unnecessary hospital visits, curb excess NHI expenditure, and improve TB outcomes.

The high number of uncomplicated cases in outpatient care at secondary care might cause unnecessary spending on NHI. The referral behavior results represent a characteristic of overtreatment of uncomplicated TB in expensive hospital outpatient departments and low back-referral (secondary care returns the patient to primary care). This phenomenon supports a study that case-based payments (i.e., Indonesian Case Base Groups, or INA-CBGs) encouraged hospitals to retain TB patients in secondary care rather than refer uncomplicated TB to the referring primary care facilities (5). Additionally, treating uncomplicated TB patients in the secondary level might not be efficient since the unit cost of secondary care is likely to be higher due to more advanced technologies and higher salaries of pulmonary specialists. Moreover, the open-ended payment system of INA-CGBs leaves open the possibility of up-coding (33) and requiring excessive visits by patients to increase revenue (41) which can lead to an unnecessarily high cost-per-episode of TB care.

Moving forward, based on the aforesaid existing studies and our study, we propose the following: the modification of payment mechanism by applying a fee-for-service for TB diagnostic (such as Xray, GeneXpert testing, smear test, and tuberculin test) and episode-based payment with giving a reward for the successful TB treatment. We suggest providing fee-for-service charge for the diagnosis to further incentivize primary care facilities (42) to participate in TB service delivery, including notifying TB cases (43). In the proposed model, NHI will cover Xray test and sputum test with microscopy, and tuberculin test through fee-for-service. While the GeneXpert will be covered as current scenario (status quo) through fee-for-service by national budget and Global Funds grants. At the treatment phase, several components of services are involved; (a) follow-up test at 2nd month (using microscopy test), transportation cost for LTFU, DM test and HIV test, paid in the first installment—at the end of intensive phase; (b) follow-up test at 5th month and end of treatment, transportation cost for LTFU, success fee for treatment completion, paid in the second installment—at the end of treatment. An episode-based payment is proposed to be paid through NHI at these stages.

We suggest establishing a clear link between provider payment, case notification, and successful TB treatment, aligning incentives to reduce unnecessary referrals. Our findings indicate that capitation-based payments at primary care facilities can discourage TB case management at the primary level, contributing to higher referral rates and additional costs. Paying the same rates for uncomplicated TB at both primary and secondary care could incentivize primary care providers to manage cases directly. As demonstrated by Taiwan’s experience, pay-for-performance (P4P) mechanisms successfully decreased default rates, increased cure rates, and reduced treatment duration (44, 45). Conversely, discontinuing P4P in Egypt led to negative behavioral changes among providers (46), underscoring the importance of sustained financial incentives. In addition to revising payment mechanisms, the government should strengthen public health support—such as TB notification and adherence assistance—and enforce contracting arrangements that include private providers. These measures, grounded in our study’s evidence of cost inefficiencies and high referral rates, would help ensure effective TB case management at the primary level while maintaining service quality and controlling costs.

This study has several limitations. First, while the national claims dataset offers broad coverage of services, they may not capture TB services delivered outside the JKN system (e.g., donor-funded or out-of-pocket), potentially underestimating service use. The study also relies on a 1% sample of NHI claims (2015–2016), which may not reflect the current TB landscape. Since primary care acts as a gatekeeper within JKN, there is an expectation that a significant number of TB patients would be found in primary care. However, our findings indicate the opposite. This discrepancy could be due to how the data were coded, the use of varied diagnosis codes, and insufficient data recording for TB patients. Also, because the claims data lack TB-treatment-outcome fields, we cannot determine whether patients adhered to—or completed—the full course of therapy.

It is also important to note that the selection of the five provinces with facility as unit of analysis may not necessarily be sufficient to provide a comprehensive picture of the TB situation in Indonesia, which consists of more than 34 provinces. However, the selection of nine districts within the five provinces aimed to capture a sufficient breadth of information while balancing with activity budget constraints and to sufficiently align with the timelines of the political process to develop policy options. Future studies could consider expanding the geographic coverage to include other provinces and districts to obtain a more representative picture of the TB situation in Indonesia. This study predominantly centered on the incentives influencing healthcare providers, while during the study we also found patient-driven referrals. Nonetheless, to augment the depth and breadth of the findings, it is recommended that future research endeavors consider the incorporation of the patient’s perspective within the qualitative study. By incorporating the viewpoints of both providers and patients, researchers can obtain a more comprehensive understanding of the subject matter, thus enriching the overall analysis. Additionally, the absence of formal validation from BPJS-K for the data analysis results may be a limitation of this study.

## 5 Conclusion

Our analysis utilizes a mixed quantitative and qualitative method and indicated that primary care facilities tend to avoid treating TB cases and tend to refer uncomplicated TB patients to secondary care. Among other factors, referrals are influenced by low capitation and provider preference for profitable diseases, such as NCDs. This behavior led to the overtreatment of uncomplicated TB in expensive hospital outpatient departments. Thus, we suggest that the government apply other payment mechanisms that can motivate the primary care facilities to provide high-quality TB service and improve national TB outcomes. Furthermore, this study calls for improved primary care’s capacity and payment mechanism change by applying fee-for-service for TB diagnosis and notification and episode-based payment for TB treatment to create stronger incentives for providers to avoid excessive use of secondary care and provide more efficient delivery of high-quality, cost-effective services in primary care would achieve more value for money for TB care in Indonesia.

## Data Availability

The datasets presented in this study can be found in online repositories. The names of the repository/repositories and accession number(s) can be found at: https://bpjs-kesehatan.go.id/.
