# Supplementary material for: The influence of provider payment mechanisms on TB service provider behavior in Indonesia: insights from National Health Insurance data and provider perspectives
Source: Front Public Health. 2025 Jul 9;13:1396596. doi: 10.3389/fpubh.2025.1396596 (PMC12283726; doi:10.3389/fpubh.2025.1396596)
Supplement: Supplementary file 1 [file Supplementary_file_1.docx]

Supplementary Material

**Understanding TB Service Delivery in Indonesia: A Descriptive Analysis of National Health Insurance Data and Qualitative Perspectives from Providers**

**Meghan O'Connell**, **Firdaus Hafidz**^*^**, Sarah Saragih** **, Cheryl Cashin, Aditia Nugroho, Laurel Hatt, Yuli Farianti, Ackhmad Afflazier, Imran Pambudi**

*** Correspondence:**
Firdaus Hafidz
[hafidz.firdaus@ugm.ac.id](mailto:hafidz.firdaus@ugm.ac.id)

# Supplementary Data

**SUPPLEMENTARY A**

**SAMPLING**

1. Sampling frame of family. List of 73,441,160 families were used based on 31^st^ December 2016 data.
2. Strata development. Combination of two variables, include PHC (22,024 facilities) and family (three categories) were utilized to develop the strata. Three categories of family consist of 1) family that never utilize health services; 2) family that utilized PHC; 3) family that utilized PHC and hospitals. If all PHC have all the three categories, 3 x 22,024 = 66,027 strata.
3. Stratified random sampling to choose family sample. 10 families were chosen in each strata, or all families if have less than 10 families in the strata. This process generated 586,969 families.
4. Generate sample data by individual membership. Membership sample data were generated from master file via family code (step 3 result). This step generated total of 1,697,452 individual sample membership.
5. Generate sample data of services by type of health facility (i.e. PHC and hospitals). Health services were retrieved from master file using membership code that were chosen in step 4.

Sampling weight. An individual weight variable was generated to ensure sample can represent population characteristic in the analysis. As described, stratified random sampling was utilized and sample was not chosen proportionally, thus there are variation of individual weight. The probability, $p$ of family, $i$, is estimated to generate family weight, $w_{i}$.

$$w_{i}=\frac{1}{p_{i}}$$

For example, strata number 3 (PHC 1 and category 3) with 6,200 family and 20 families were chosen. Each family has a probability of 20/6,200 to be chosen. Sample family weight is 6,200/ 20 = 310. One family represent 310 family in the population. Variation of population in each strata causes variation of weight in different strata. Next, individual weight was estimated as the ratio of family weight and number of family member. Table 1 illustrate the calculation of family and individual weight.

*Table 1 Illustration of family weight in six strata*

| No | PHC code | Family category | Number of family | Number of sample | Probability, $p_{i}$ | Family weight, $w_{i}$ | Number of family member | Individual weight |
| --- | --- | --- | --- | --- | --- | --- | --- | --- |
| 1 | 1 | 1 | 150 | 20 | 0,133 | 7.5 | 3 | 2.5 |
| 2 | 1 | 2 | 400 | 20 | 0,050 | 20 | 4 | 5.0 |
| 3 | 1 | 3 | 6200 | 20 | 0,003 | 310 | 5 | 62.0 |
| 4 | 2 | 1 | 200 | 20 | 0,100 | 10 | 4 | 2.5 |
| 5 | 2 | 2 | 500 | 20 | 0,040 | 25 | 5 | 5.0 |
| 6 | 2 | 3 | 5900 | 20 | 0,0003 | 295 | 3 | 98.3 |

Five main files were generated: 1) Individual sample dataset; 2) Health services at PHC (capitation), 3) Health services at PHC (non-capitation), 4) Hospital services and 5) Secondary diagnosis sub-set. There are 1,697,452 unique list of individual data, consist of demographic characteristics (e.g. age, sex, marital status) and membership information (e.g. health facility registered, segment). More than 1.7 million of PHC services (capitation) visit, 104 thousand of non-capitation visits, and 906 thousand visits at hospital with 700 thousand secondary diagnosis observation. Health services dataset include health facility characteristics (e.g. type, ownership), diagnosis, and claim of the services.

**Data and Variables**

Here is the list of variables utilized in analysis:

| No. | Variable | Description | Variable constructed and assumption |
| --- | --- | --- | --- |
| 1 | PSTV01 | Unique individual ID |  |
| 2 | PSTV03 | Date of birth | Age by 31^st^ of December 2016 |
| 3 | PSTV04 | Family membership |  |
| 4 | PSTV05 | Sex |  |
| 5 | PSTV06 | Marital status |  |
| 6 | PSTV08 | Membership segment |  |
| 7 | PSTV09 | Province |  |
| 8 | PSTV12 | Health facility registered |  |
| 9 | PSTV15 | Individual weight |  |
| 10 | FKP03 | Date visit at PHC |  |
| 11 | FKP04 | Date discharge at PHC |  |
| 12 | FKP09 | Type of PHC |  |
| 13 | FKP10 | Type of services at PHC |  |
| 14 | FKP13 | Discharge status |  |
| 15 | FKP14 | Diagnosis at PHC | Generate new variable to identify tuberculosis diagnosis with inclusion criteria of "tuberculosis" and exclude "Z23.2 Need for immunization against tuberculosis (BCG)" |
| 16 | PNK03 | Visit date at PHC (non-capitation) |  |
| 17 | PNK05 | Discharge date at PHC (non-capitation) |  |
| 18 | PNK10 | Type of PHC |  |
| 19 | PNK11 | Type of services at PHC (non capitation) |  |
| 20 | PNK13 | Diagnosis at PHC (non-capitation) | Generate new variable to identify tuberculosis diagnosis with inclusion criteria of "tuberculosis" and exclude "Z23.2 Need for immunization against tuberculosis (BCG)" |
| 21 | PNK18 | Claim verified |  |
| 22 | FKL03 | Visit date at hospital |  |
| 23 | FKL04 | Discharge date at hospital |  |
| 24 | FKL09 | Type of hospitals |  |
| 25 | FKL10 | Type of services at hospital |  |
| 26 | FKL14 | Discharge status |  |
| 27 | FKL15 | Primary diagnosis | Generate new variable to identify tuberculosis diagnosis with inclusion criteria of "tuberculosis" and exclude "Z23.2 Need for immunization against tuberculosis (BCG)" |

**Classification of Uncomplicated TB**

**Uncomplicated TB Diagnosis at Primary Care Facilities**

- A157 Primary respiratory tuberculosis confirmed bacteriologically and histologically
- A158 Other respiratory tuberculosis, confirmed bacteriologically and histologically
- A159 Respiratory tuberculosis unspecified confirmed bacteriologically and histologically
- A167 Primary respiratory tuberculosis without mention of bacteriological or histological confirmation
- A168 Other respiratory tuberculosis, without mention of bacteriological or histological confirmation
- A169 Respiratory tuberculosis unspecified, without mention of bacteriological or histological confirmation
- B909 Sequelae of respiratory and unspecified tuberculosis
- Z201 Contact with and exposure to tuberculosis

**Uncomplicated Primary Diagnosis at Secondary Care**

- J209 Acute bronchitis, unspecified
- J00 Acute nasopharyngitis [common cold]
- J029 Acute pharyngitis, unspecified
- J159 Bacterial pneumonia, unspecified
- J40 Bronchitis, not specified as acute or chronic
- Z509 Care involving the use of rehabilitation procedure, unspecified
- Z548 Convalescence following other treatment
- Z549 Convalescence following unspecified treatment
- R05 Cough
- Z719 Counselling unspecified
- Z719 Counselling, unspecified
- R42 Dizziness and giddiness
- K30 Dyspepsia
- Z048 Examination and observation for other specified reasons
- Z049 Examination and observation for an unspecified reason
- Z011 Examination of ears and hearing
- Z833 Family history of diabetes mellitus
- Z836 Family history of diseases of the respiratory system
- Z824 Family history of ischemic heart disease and other diseases of the circulatory system
- Z831 Family history of other infectious and parasitic diseases
- R509 Fever, unspecified
- Z088 Follow-up examination after other treatment for malignant neoplasm
- Z098 Follow-up examination after other treatment for other conditions
- Z094 Follow-up examination after treatment of fracture
- Z089 Follow-up examination after unspecified treatment for malignant neoplasm
- Z099 Follow-up examination after unknown treatment for other conditions
- R51 Headache
- E785 Hyperlipidemia, unspecified
- Z289 Immunization not carried out for an unspecified reason
- T814 Infection following a procedure, not elsewhere classified
- Z760 Issue of repeat prescription
- Z017 Laboratory examination
- Z038 Observation for other suspected diseases and conditions
- Z039 Observation for suspected disease or condition, unspecified
- R104 Other and unspecified abdominal pain
- R268 Corrosion of other parts of eye and adnexa
- B99 Other and unspecified infectious diseases
- Z008 Other general examinations
- A158 Other respiratory tuberculosis, confirmed bacteriologically and histologically
- A168 Other respiratory tuberculosis, without mention of bacteriological or histological confirmation
- Z718 Other specified counselling
- K318 Other specified diseases of stomach and duodenum
- Z518 Other specified medical care
- Z018 Other specified special examinations
- M478 Other spondylosis
- R529 Pain, unspecified
- Z751 Person awaiting admission to an adequate facility elsewhere
- Z870 Personal history of diseases of the respiratory system
- Z861 Personal history of infectious and parasitic diseases
- Z929 Personal history of medical treatment, unspecified
- Z928 Personal history of other medical treatment
- A157 Primary respiratory tuberculosis confirmed bacteriologically and histologically
- A167 Primary respiratory tuberculosis without mention of bacteriological or histological confirmation
- A159 Respiratory tuberculosis unspecified confirmed bacteriologically and histologically
- A169 Respiratory tuberculosis unspecified, without mention of bacteriological or histological confirmation
- B909 Sequelae of respiratory and unspecified tuberculosis
- A160 Tuberculosis of the lung, bacteriologically and histologically negative
- A150 Tuberculosis of the lung, confirmed by sputum microscopy with or without culture
- A153 Tuberculosis of the lung, confirmed by unspecified means
- A162 Tuberculosis of the lung, without mention of bacteriological or histological confirmation
- J22 Unspecified acute lower respiratory infection
- M1090 Gout, unspecified, multiple sites
- M1098 Gout, unspecified, other sites

**Uncomplicated secondary diagnosis at Hospitals**

- A16 Respiratory tuberculosis, not confirmed bacteriologically or histologically
- A15 Respiratory tuberculosis, bacteriologically and histologically confirmed
- B90 Sequelae of tuberculosis
- J40 Bronchitis, not specified as acute or chronic
- J18 Pneumonia, organism unspecified
- J06 Acute upper respiratory infections of multiple and unspecified sites
- R50 Fever of other and unknown origin
- J20 Acute bronchitis
- Z01 Other special examinations and investigations of persons without complaint or reported diagnosis
- B99 Other and unspecified infectious diseases
- J98 Other respiratory disorders
- R62 Lack of expected normal physiological development
- J00 Acute nasopharyngitis [common cold]
- R51 Headache
- Z03 Medical observation and evaluation for suspected diseases and conditions ruled out
- Z11 Special screening examination for infectious and parasitic diseases
- R11 Nausea and vomiting
- Z09 Follow-up examination after treatment for conditions other than malignant neoplasms
- K02 Dental caries
- R63 Symptoms and signs concerning food and fluid intake
- G44 Other headache syndromes
- Z86 Personal history of certain other diseases
- R53 Malaise and fatigue
- R05 Cough
- Z87 Personal history of other diseases and conditions
- J02 Acute pharyngitis
- Z71 Persons encountering health services for other counselling and medical advice, not elsewhere classified
- R42 Dizziness and giddiness
- Z02 Examination and encounter for administrative purposes
- A91 Dengue hemorrhagic fever
- Z76 Persons encountering health service
