# Supplementary material for: The influence of provider payment mechanisms on TB service provider behavior in Indonesia: insights from National Health Insurance data and provider perspectives
Source: Front Public Health. 2025 Jul 9;13:1396596. doi: 10.3389/fpubh.2025.1396596 (PMC12283726; doi:10.3389/fpubh.2025.1396596)
Supplement: Supplementary file 2 [file Supplementary_file_2.docx]

Supplementary Material

**Understanding TB Service Delivery in Indonesia: A Descriptive Analysis of National Health Insurance Data and Qualitative Perspectives from Providers**

**Meghan O'Connell**, **Firdaus Hafidz**^*^**, Sarah Saragih** **, Cheryl Cashin, Aditia Nugroho, Laurel Hatt, Yuli Farianti, Ackhmad Afflazier, Imran Pambudi**

*** Correspondence:**
Firdaus Hafidz
[hafidz.firdaus@ugm.ac.id](mailto:hafidz.firdaus@ugm.ac.id)

# Supplementary Data

**Consolidated criteria for reporting qualitative studies (COREQ): 32-item checklist**

Developed from:

Tong A, Sainsbury P, Craig J. Consolidated criteria for reporting qualitative research (COREQ): a 32-item checklist for interviews and focus groups. *International Journal for Quality in Health Care*. 2007. Volume 19, Number 6: pp. 349 – 357

| **No. Item** | **Guide questions/description** | **Reported on Page #** |
| --- | --- | --- |
| **Domain 1: Research team and reﬂexivity** |  |  |
| *Personal Characteristics* |  |  |
| 1. Inter viewer/facilitator | Which author/s conducted the interview or focus group? | The interviews were conducted by FH (male, MD, PhD, research consultant), MO (female, PhD, research consultant), SS (female, MSc, research consultant), CC (female, PhD, research consultant), AN (male, PhD, research consultant) LH (female, PhD, research consultant), YF (female, MD, M.Epid, Government MoH officer), AA (male, MD, MKM, Government MoH officer), IP (male, MD, MPHM, Government MoH officer) |
| 2. Credentials | What were the researcher’s credentials? E.g. PhD, MD  The first author/researcher credential is MD and PhD, the rest of the researchers’ credentials are MD, PhD, MPH, M.Epid, and MHPM |  |
| 3. Occupation | What was their occupation at the time of the study? |  |
| 4. Gender | Was the researcher male or female?  Four of the researchers are males and five of the researchers are females |  |
| 5. Experience and training | What experience or training did the researcher have? | The interviewers either had experience in qualitative research data acquisition and/or experiences as principal investigators of qualitative studies. In addition all interviewers were provided with a focus group interview guide, including transcription rules to collect data. |
| *Relationship with participants* |  |  |
| 6. Relationship established | Was a relationship established prior to study commencement?  No | - |
| 7. Participant knowledge of the interviewer | What did the participants know about the researcher? e.g. personal goals, reasons for doing the research  None of the above | - |
| 8. Interviewer characteristics | What characteristics were reported about the interviewer/facilitator? e.g. Bias, assumptions, reasons and interests in the research topic  None of the above | - |

| **Domain 2: study design** |  |  |
| --- | --- | --- |
| *Theoretical framework* |  |  |
| 9. Methodological orientation and Theory | What methodological orientation was stated to underpin the study? e.g. grounded theory, discourse analysis, ethnography, phenomenology, content analysis | 3-5 |
| *Participant selection* |  |  |
| 10. Sampling | How were participants selected? e.g. purposive, convenience, consecutive, snowball | 5 |
| 11. Method of approach | How were participants approached? e.g. face-to-face, telephone, mail, email | 7 |
| 12. Sample size | How many participants were in the study? | 5 |
| 13. Non-participation | How many people refused to participate or dropped out? Reasons? | 6,7 |
| *Setting* |  |  |
| 14. Setting of data collection | Where was the data collected? e.g. home, clinic, workplace | 7 |
| 15. Presence of non-participants | Was anyone else present besides the participants and researchers? | 7 |
| 16. Description of sample | What are the important characteristics of the sample? e.g. demographic data, date | - |
| *Data collection* |  |  |
| 17. Interview guide | Were questions, prompts, guides provided by the authors? Was it pilot tested? | 7 |
| 18. Repeat interviews | Were repeat interviews carried out? If yes, how many?  No repeat interview | - |
| 19. Audio/visual recording | Did the research use audio or visual recording to collect the data? | 7 |
| 20. Field notes | Were ﬁeld notes made during and/or after the interview or focus group? | 7 |
| 21. Duration | What was the duration of the interviews or focus group? | 7 |
| 22. Data saturation | Was data saturation discussed?  Saturation is defined as no new codes in subsequent FGD. | - |
| 23. Transcripts returned | Were transcripts returned to participants for comment and/or correction? | 7 |
| **Domain 3: analysis and ﬁndings** |  |  |
| *Data analysis* |  |  |
| 24. Number of data coders | How many data coders coded the data?  One data coder, SM | - |
| 25. Description of the coding tree | Did authors provide a description of the coding tree?  No | - |
| 26. Derivation of themes | Were themes identiﬁed in advance or derived from the data?  No | - |
| 27. Software | What software, if applicable, was used to manage the data?  Yes, excel | - |
| 28. Participant checking | Did participants provide feedback on the ﬁndings?  No | - |
| *Reporting* |  |  |
| 29. Quotations presented | Were participant quotations presented to illustrate the themes/ﬁndings? Was each quotation identiﬁed? e.g. participant number | Quotations are presented in the results section Each quotation was indexed using the name of the institution/provider and number. Eg: Private clinic 2, private clinic 4 |
| 30. Data and ﬁndings consistent | Was there consistency between the data presented and the ﬁndings? | We endeavored to ensure consistency between the data presented and the findings by using quotes to support our interpretations/findings. |
| 31. Clarity of major themes | Were major themes clearly presented in the ﬁndings? | We described our findings including original quotes from the participants in the results section. |
| 32. Clarity of minor themes | Is there a description of diverse cases or discussion of minor themes? | We described our findings including original quotes from the participants in the results section. |
